# Biological Chemistry of Hydrogen Selenide

**DOI:** 10.3390/antiox5040042

**Published:** 2016-11-22

**Authors:** Kellye A. Cupp-Sutton, Michael T. Ashby

**Affiliations:** Department of Chemistry and Biochemistry, University of Oklahoma, Norman, OK 73019, USA; kellye.a.cupp-1@ou.edu

**Keywords:** biological reactive selenium species, hydrogen selenide, selenocysteine, selenomethionine, selenosugars, selenophosphate, selenocyanate, selenophosphate synthetase thioredoxin reductase

## Abstract

There are no two main-group elements that exhibit more similar physical and chemical properties than sulfur and selenium. Nonetheless, Nature has deemed both essential for life and has found a way to exploit the subtle unique properties of selenium to include it in biochemistry despite its congener sulfur being 10,000 times more abundant. Selenium is more easily oxidized and it is kinetically more labile, so all selenium compounds could be considered to be “Reactive Selenium Compounds” relative to their sulfur analogues. What is furthermore remarkable is that one of the most reactive forms of selenium, hydrogen selenide (HSe^−^ at physiologic pH), is proposed to be the starting point for the biosynthesis of selenium-containing molecules. This review contrasts the chemical properties of sulfur and selenium and critically assesses the role of hydrogen selenide in biological chemistry.

## 1. Overview of Chalcogens in Biology

Chalcogens are the chemical elements in group 16 of the periodic table. This group, which is also known as the oxygen family, consists of the elements oxygen (O), sulfur (S), selenium (Se), tellurium (Te), and the radioactive element polonium (Po). O, S, and Se are essential for life, although Se is only required in trace amounts of about 15 mg in a typical 70 kg adult human [1]. The conventional roles of oxygen in aerobic respiration and photosynthesis (O_2_), water (~65 wt % of the human body), and a myriad of oxygen-containing bioorganic molecules (amino acids, nucleotides, etc.), may be contrasted with the sometimes deleterious properties of biological reactive oxygen species (ROS: OH•, O_2_^−^•, H_2_O_2_, etc.) [2,3,4]. Similarly, sulfur serves a conventional role in the amino acids (e.g., Cys and Met) and in biological reactive sulfur species (RSS: H_2_S, OSCN^−^ [5,6], polysulfides, etc.) [7,8,9,10,11,12,13]. Although it is toxic in large doses, selenium is an essential micronutrient for animals [14,15,16,17]. In plants, it sometimes occurs in toxic amounts as forage, e.g., locoweed [18]. Selenium is a component of the amino acids selenocysteine (Sec, the “21st amino acid”) [19] and selenomethionine (SeM) [20]. In humans, according to proteomics, Sec is incorporated into at least 25 different proteins [21,22,23]. The identified proteins, including glutathione peroxidases and certain forms of thioredoxin reductase, incorporate Sec into their active sites, whereas SeM is randomly incorporated into proteins [21,22,24,25,26]. As selenium compounds are generally more reactive than the corresponding sulfur derivatives (*vide infra*), one might argue that all biologically relevant selenium compounds are “Reactive Selenium Species”. The present review focuses on hydrogen selenide (H_2_Se), typically the least stable oxidation state for selenium in a biological setting and the putative reactant that leads to all known selenium-containing biomolecules.

## 2. Chemistry of Sulfur vs. Selenium

Sulfur is approximately 10^5^ times more abundant in the human body than selenium, but the latter element is selected for certain biological functions [27]. It is particularly remarkable that this is achieved, as there are no other two main-group elements that exhibit more similar physical and chemical properties [28]. Nonetheless, the selective use of selenium must eventually rely upon the unique chemical properties of the element. Despite the fact that the average atomic mass of Se is more than twice that of S, the atomic radii are remarkable similar, as a consequence of d-block contraction [29], as are the bond lengths that are observed in main-group compounds (Table 1). Furthermore, the electronegativities of the two elements are comparable, and both are more similar to carbon (χ = 2.55) than any other elements of the periodic table. However, important differences exist between the two elements. S forms stronger covalent bonds relative to Se (Table 1). Additionally, it is easier to oxidize Se than S (Table 1). Moreover, selenols are more acidic than thiols with acid dissociation constants that are typically 3–4 orders of magnitude larger (Table 1).

### 2.1. Selenium Compounds Are More Reactive

Given that free energy relationships frequently exist between thermodynamics and kinetics [49,50], it is unsurprising that Se compounds tend to be more reactive than S compounds. For example, for the exchange of thiols and selenolates (note the proton state of each [51]) with disulfides, diselenides, and mixed chalcogenides, reaction rates of selenium as a nucleophile and as an electrophile are 2–3 and 4 orders of magnitude higher, respectively, than those of sulfur *at neutral pH* [52]:

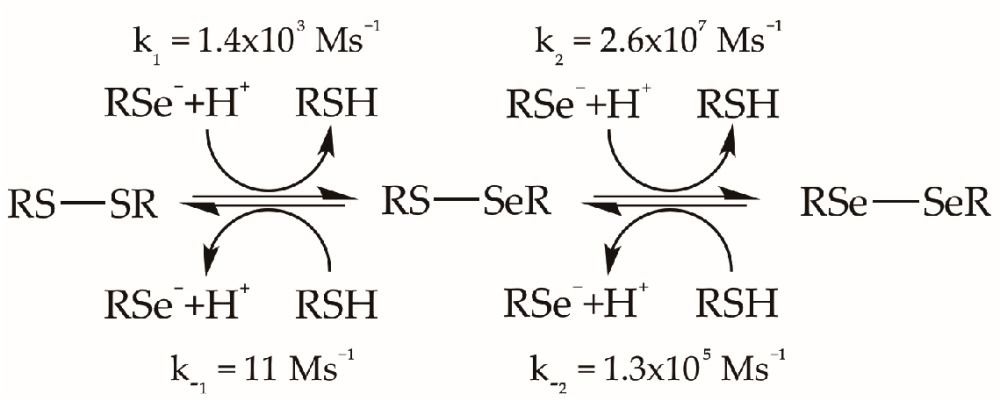
(1)

(2) $$\mathrm{RSSR}+2{\mathrm{RSe}}^{-}+2H^{+}⇌\mathrm{RSeSeR}+2\mathrm{RSH}$$

From left to right, the greater nucleophilicity of Sec relative to Cys may be attributed to the fact that Se is deprotonated and S is protonated at neutral pH, and to the fact that a S–H covalent bond is formed when selenolate reacts and Cys is liberated. In the other direction, the greater electrophilicity of Se in this particular reaction is attributed to properties of the S–S vs. Se–Se vs. Se–S bonds, and not the nature of the leaving group. From these rate constants, the ratio of cystine (RSSR) to selenocystine (RSeSeR) is given by: K = K_1_ × K_2_ = k_1_ × k_2_/k_−1_ × k_−2_ = 2.5 × 10^4^ for [Cys] = [Sec]. While this result may at first appear inconsistent with the X–X bond strengths of Table 1, the equilibrium of Equation (2) in fact reflects a very important difference between S and Se, selenolates are more stable than thiolates, as reflected in the pK_a_ values of Table 1:

(3) $$\mathrm{RSeH}⇌{\mathrm{RSe}}^{-}+H^{+},K_{a}\left(\mathrm{Se}\right)=10^{-5.43}$$

(4) $${\mathrm{RS}}^{-}+H^{+}⇌\mathrm{RSH},\;1/K_{a}\left(S\right)=10^{8.22}$$

(5) $$\bar{{\mathrm{RSeH}\;+\;\mathrm{RS}}^{-}\;⇌{\;\mathrm{RSe}}^{-}{\;+\;\mathrm{RSH},\;K}_{a}\left(\mathrm{Se}\right){/K}_{a}\left(S\right)\;=\;617}$$

The difference in pK_a_ between Cys and Sec (Equation (5)) drives the formation of the unfavorable Se–Se bond at pH 7. This difference in pK_a_ is largely due to the greater polarizability (not electronegativity) of Se [40,41] that delocalizes charges and stabilizes the selenolate (Table 1). The example of Equations (3)–(5) illustrates how subtle differences in the chemical properties between S and Se can be exploited to drive otherwise unfavorable reactions.

### 2.2. Selenium Favors Higher Oxidation States

**Figure 1:**
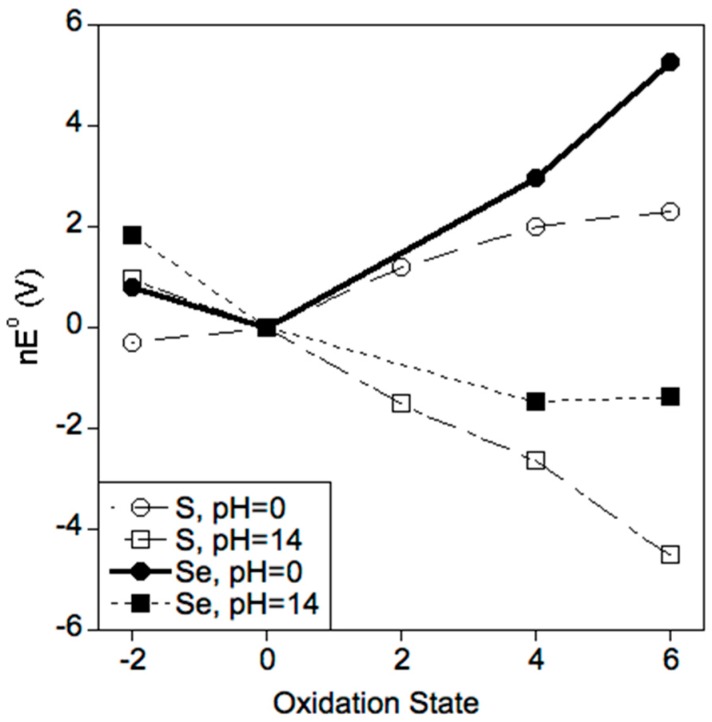
Frost diagrams for sulfur and selenium at pH 0 and 14. The nE° = 0 value is arbitrarily assigned to the zero-valent state. Note that S(−II) and Se(0) are the most stable oxidation states at pH 0, and that S(VI) and Se(VI) are the most stable at pH 14.

The ionization energies of Table 1 suggest Se is easier to oxidize than S. This fact is further evidenced in the Frost diagrams of Figure 1 [53,54]. Thus, for a particular pH (0 or 14) and a given oxidation state (−II to +VI), Se is more easily oxidized than S. Note the general trend that oxidation potentials increase with oxidation states at low pH and they decrease with oxidation state at high pH. These trends are due to the effects of the proton states of the species involved. Finally, note that the most stable oxidation state at pH 0 for S and Se are −II and 0, respectively.

## 3. Selenium in Biology

Se is an essential trace element and a potential toxin. It is the only trace element found in proteins that is genetically encoded. Selenocysteine (Sec) [19], the “21st amino acid”, is incorporated into selenoproteins by a co-translational process that uses translation machinery that redefines UGA (uracil/guanine/Adenine) codons to encode Sec [56,57]. Many biological functions are performed by selenoproteins, all of which contain Sec residues in their primary structures [58]. The roles of Sec in proteins has been thoroughly reviewed [21,23,24,59,60].

Although an essential trace element, Se is toxic if taken in excess. Exceeding the Tolerable Upper Intake Level (UL) of 400 micrograms per day can lead to selenosis [61]. Selenomethionine (SeM), selenocysteine (Sec), selenite (SeO_3_^2−^), and selenate (SeO_4_^2−^) account for almost all the Se in diets, and all these forms are absorbed without regulation. However, SeM is the principal chemical form of Se in most mammalian diets. It is synthesized by plants and incorporated randomly into plant proteins at Met positions. Once absorbed, SeM is transported and incorporated randomly into mammalian proteins using the Met system.

### 3.1. Which Oxidation States of Sulfur and Selenium Are (Most) Stable in Biology?

**Figure 2:**
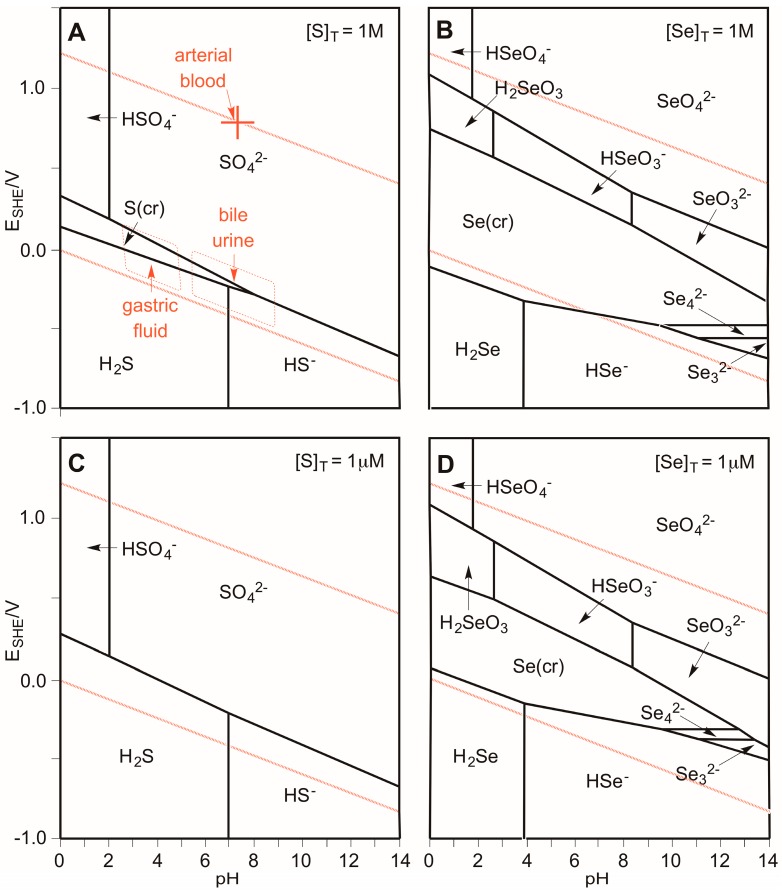
Pourbaix diagrams for S (**A** and **C**) and Se (**B** and **D**) at 1M total chalcogen (**A** and **B**) and 1 μM total chalcogen (**C** and **D**) at 25 °C. The red lines define the stability region of water. Various physiological fluids are illustrated (**A**) [55].

Figure 2 illustrates the Pourbaix diagrams for S and Se [62]. The diagrams describe the thermodynamically preferred oxidation states of the elements as a function of applied potential and pH [63,64,65]. Note these propensity diagrams are concentration-dependent [66]. Two examples for each element are illustrated: standard conditions (top, total concentration of chalcogen equal to 1 M), and more dilute conditions (bottom, 1 μM). The dashed red lines encompass the stability region of water. Potentials above the top line would oxidize water to produce O_2_, and potentials below the bottom line would reduce water to produce H_2_. Any species that overlaps the stability region of water would be thermodynamically stable under the E/pH conditions of the overlap. For S at high concentrations (top left), the −II, 0, and +VI oxidations states are stable, although S(0) disproportionate to S(−II) and S(+IV) under alkaline conditions. However, at lower concentrations of S, only the −II and +VI oxidations states are thermodynamically stable (bottom left). In contrast to S, Se in the −II, 0, +IV, and +VI oxidations states are stable in water, although H_2_Se is capable of reducing water at high concentrations under acidic conditions (top right), but not once the H_2_Se is sufficiently diluted (bottom right). We can conclude that selenide is thermodynamically stable with respect to water under anaerobic conditions, but what about oxygen-rich environments? Selenide solutions above 10^−6^ M are known to be oxidized under atmospheric pressures of oxygen in less than three minutes at pH 7 [67]. Air-saturated water contains 2.7 × 10^−4^ M dissolved O_2_. Since it has been determined that the reaction is zero-order in oxygen for [O_2_] > 10^−4^ M and first-order in [H_2_Se], we can conclude that the half-life for oxidation of selenide to colloidal elemental selenium in air-saturated water (in the absence of other reactants) is about 30 s at pH 7, and that the rate is independent of the concentration of selenide [67]. By comparison, solutions of H_2_S are oxidized over a period of several hours under similar conditions.

Three important conclusions can be drawn from Figure 1 and Figure 2: (1) Se is more easily oxidized relative to S (Figure 1); (2) Se exhibits more stable oxidation states in water relative to S (Figure 2); and (3) Se(0) and S(+VI) are the most thermodynamically stable oxidation states for Se and S, respectively, in an aqueous environment in the absence of an applied SHE potential (Figure 1 and Figure 2). For tissues that are perfused with arterial blood at pH 7.37 (an equivalent potential of +0.782 V), the thermodynamically most stable oxidation state for S and Se at all concentrations is +VI (Figure 2). In contrast, the −II oxidation state becomes more stable under the reducing conditions of gastric fluid, bile, and urine (Figure 2). Importantly, this discussion of thermodynamics does not address the issue of whether a given reaction is kinetically viable. Indeed, biological redox systems are typically not in thermodynamic equilibrium [68]. For example, the thiol and disulfide forms of glutathione (GSH in its reduced and GSSG in its oxidized forms) and cysteine (Cys in its reduced and CySSCy in its oxidized forms) that are measured in human plasma suggest that the GSSG/2GSH and the CySSCy/2Cys pools are not in redox equilibrium [69].

Note that in many biological settings, H_2_Se is thermodynamically unstable, for example, in the oxygenated intercellular fluids of eukaryotic cells. However, those cells contain millimolar concentrations of GSH, most of which is kept in the reduced state by glutathione reductase. Furthermore, SeO_3_^2−^ is known to react spontaneously with GSH [70,71,72,73,74] to initially produce selenodiglutathione (GS-Se-SG), Equation (6). In the presence of excess GSH, GSSeSG is further reduced to glutathioselenol (GSSeH), Equation (7). GSSeH either spontaneously dismutates into Se(0) and GSH, Equation (8), or is further reduced by GSH to yield H_2_Se, Equation (9). As discussed before, H_2_Se is readily oxidized by O_2_ into Se(0), Equation (10).

(6) $${\mathrm{SeO}}_{3}^{2^{-}}+4\mathrm{GSH}+2H^{+}\to \mathrm{GSSeSG}+\mathrm{GSSG}+3H_{2}O$$

(7) GSSeSG+GSH→GSSeH+GSSG

(8) GSSeH→Se(0)+GSH

(9) $$\mathrm{GSSeH}+\mathrm{GSH}\to H_{2}\mathrm{Se}+\mathrm{GSSG}$$

(10) $$H_{2}\mathrm{Se}+1/2O_{2}\to \mathrm{Se}\left(0\right)+H_{2}O$$

However, excess GSH can protect H_2_Se from oxidation. Accordingly, while Se(−II) may be thermodynamically unstable in a biological setting, it may be kinetically stabilized, Equation (11).

(11) $$H_{2}\mathrm{Se}+1/2O_{2}+2\mathrm{GSH}\to H_{2}\mathrm{Se}+\mathrm{GSSG}+H_{2}O$$

Indeed, it is precisely the role of GSH to protect functional groups from oxidation.

### 3.2. Role of Selenide In Vivo

Before discussing H_2_Se, it is valuable to review the mechanisms by which its S congener is released in vivo: (1) H_2_S is immediately released after its production by enzymes (e.g., cystathionine β-synthase [75], cystathionine γ-lyase [76], 3-mercaptopyruvate sulfurtransferase [77], and cysteine aminotransferase [78]); and (2) it can be released via non-enzymatic processes from stored labile sulfur [79]. Two forms of sulfur stores in cells that have been identified are [80,81]: (1) acid-labile sulfur [82]; and (2) bound sulfane sulfur (RS(S)_n_^−^, which releases H_2_S under reducing conditions) [83]. Acid-labile sulfur is mainly sulfur atoms in the iron–sulfur proteins (e.g., ferredoxins), which play a critical role in a wide range of redox reactions. However, H_2_S is only released from acid-labile sulfur below pH 5.4 [82], which is well below most physiologic fluids. Like Cys, H_2_S exhibits both anti- and pro-oxidant behavior [84].

**Figure 3:**
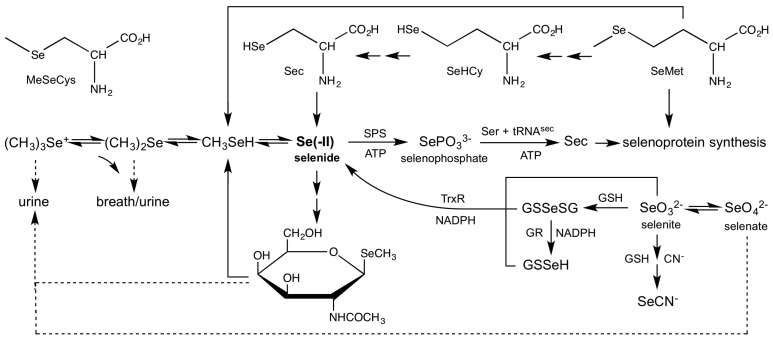
Metabolic pathways for dietary selenium compounds.

In the previous section we discussed the various oxidation states of Se that exhibit thermodynamic stability with respect to water and molecular oxygen (for certain [Se], [H^+^], and [O_2_]). However, even under conditions in which H_2_Se exhibits thermodynamic stability, it remains a powerful reductant, with a reduction potential close to that of H_2_. Not surprisingly, H_2_Se has not been directly identified in vivo. Nonetheless, it is widely held that H_2_Se exists at the intersection of various metabolic pathways (Figure 3). The principle dietary sources of selenium are SeM and SeO_3_^2−^, and both are apparently sources of H_2_Se. Two metabolic pathways have been identified that convert SeM to H_2_Se. SeM is converted to methylselenol (MeSeH) [85] by γ-lyases [86], which is subsequently demethylated to H_2_Se [87]. MeSeH is also produced from methylselenocysteine (MeSeCys) and cysteine β-lyases [88,89]. Alternatively, a transselenation pathway yields Sec, which is subsequently reduced by β-lyases to H_2_Se [90]. The reduction of SeO_3_^2−^ to H_2_Se can either be carried out directly by thioredoxin and thioredoxin reductases (TrxR) [91], or by glutathione reductases (GR) via the intermediate selenodiglutathione (GSSeSG) [92]. There is one known productive use of H_2_Se, as the source of selenium for selenophosphate synthetase (SPS) [93]. The product selenophosphate, SePO_3_^3−^, is subsequently used in the synthesis of Sec. H_2_Se can be excreted by two metabolic pathways, as selenosugars or as methylated derivatives. The most common elimination products for Se are selenium-containing carbohydrates [94] such as methyl-2-acetamido-2-deoxy-1-seleno-β-d-galactopyranoside (SeGalNAc). However, H_2_Se is eliminated after methylation in the form of dimethylselenide (Me_2_Se) via breath [95] or trimethylselenonium (Me_3_Se^+^) ion via urine [96]. We will next systematically examine the evidence for involvement of H_2_Se in each of these reactions.

### 3.3. Mechanism of Selenophosphate Synthetase (SPS)

The synthesis of Sec is the only known productive use of H_2_Se in a biological setting. The first step of the biosynthesis involves production of the intermediate SePO_3_^3−^. SPS requires some form of reduced Se (such as H_2_Se) and ATP (adenosine triphosphate) as substrates in order to generate a stoichiometric amount of SePO_3_^3−^, AMP (adenosine monophosphate), and orthophosphate [93]. Early studies by Stadtman et al. established that a SPS enzyme preparation from *Salmonella typhimurium* produced “a compound containing selenium bonded to phosphorus” only in the presence of NaSeH, MgCl_2_, PO_4_^3−^, and ATP [97,98]. The compound formed was later identified as SePO_3_^3−^ [99]. Omitting any of the components of the system eliminated production of SePO_3_^3−^, although SeO_3_^2−^ + dithiothreitol (DTT) could replace NaSeH, whereby Sec produced lesser amounts of SePO_3_^3−^ that were attributed to contamination of the Sec by NaSeH. While this is not direct detection of H_2_Se, we find these observations the most compelling evidence for its involvement in biochemistry.

### 3.4. Reduction of Inorganic Selenium

SeO_3_^2−^ and SeO_4_^2−^ are the primary environmental inorganic sources of Se. To be incorporated into amino acids, a reduction must occur from Se(IV) and Se(VI) to Se(−II), a process that involves 6 and 8 e^−^, respectively. While one- and two-electron reduction mechanisms are commonplace, it is very improbable that the elementary reaction steps involve 4–6 e^−^ processes. Consequently, Se(0) is likely to be an intermediate, albeit not necessary in elemental form. Multi-electron biochemical reductions frequently involve unstable intermediates, for example, in fixation, an 8 e^−^ process. Unstable intermediates are sometimes protected at the active sites at which they are produced, and in other cases, they are converted to relatively stable free molecules prior to further reaction. It is noteworthy that a number of derivatives of Se in the formal −II oxidation state have been identified in a biological setting. Besides H_2_Se, such species include RSeH (MeSeH [87,100], Sec [101], SeHCys [86], etc.), SePO_3_^3−^ [93], and SeCN^−^ [102]. Many of these compounds are on the pathway to/from H_2_Se, but there exists very little direct evidence that H_2_Se is involved. Nonetheless, given the significant evidence that SPS employs H_2_Se as a substrate, it has been shown that Se delivered from Sec by an *Escherichia coli* cysteine desulfurase-like protein can replace H_2_Se in the in vitro SPS assay for SePO_3_^3−^ formation [103,104]. These and related studies have demonstrated, in the presence of supporting enzymes, that Se(−II) can be mobilized from alternative sources.

### 3.5. Mechanism of Selenocysteine β-Lyase

Sec is incorporated into proteins co-translationally, as directed by the UGA codon. This is achieved by employing a special tRNA that has an anticodon complimentary to UGA (tRNA^Sec^). In many species of bacteria, this tRNA^Sec^ is first amino acylated with serine to yield seryl-tRNASec, which is subsequently converted to selenocysteyl-tRNA^Sec^. SePO_3_^3−^ serves as the Se donor in the known systems that produce selenocysteyl-tRNA^Sec^ [19]. Selenocysteine β-lyase catalyzes the chemical reaction: Sec + reduced acceptor → H_2_Se + Ala + acceptor [90,97]. The enzyme was first purified from pig liver in 1982 [90]. The reducing agent in laboratory studies is typically DTT. A subsequent, more detailed investigation of the analogous enzyme isolated from *Citrobacter feundii* suggested elemental Se(0) and not H_2_Se was produced in the absence of DTT [105]. A decade later, the nifs gene product was found to be a pyridoxal phosphate binding enzyme that catalyzes the desulfurization of Cys to yield Ala and S(0). Relevant to the present topic, as mentioned previously, these so-called NIFS proteins (or cysteine desulfurase) were found to employ Sec as a source of Se for SPS to produce SePO_3_^3−^ [103,104]. Importantly, transfer of Se from free Sec by the NIFS protein to SPS occurred in aqueous solution without equilibration with a solvent [104]. Thus, a complex between the NIFS and SPS enzymes may take place that facilitates the direct transfer of Se(−II). In addition to allowing for greater differentiation between S and Se in vivo, such a hands-off mechanism could serve to stabilize reactive intermediates (cf. nitrogenase).

### 3.6. Demethylation of Methylselenol

There are several pathways that lead to MeSeH. Once formed, MeSeH can be sequentially dimethylated to give the diselenide (Me)_2_Se, and trimethylated to give (Me)_3_Se^+^. The suggestion that H_2_Se might be an intermediate in the production of MeSeH from SeO_3_^2−^ was first made in 1966 [106]. However, the focus here is on the demethylation of MeSeH to give H_2_Se [87], which was first proposed forty years later [107,108,109]. To date, H_2_Se has not been directly observed by the demethylation of MeSeH. However, the Se liberated by rat organ supernatants and homogenates was quantified as its oxidation product SeO_3_^2−^ [107]. Without the identification of specific demethylation pathways and the exclusion of alternative processes, it will not be possible to unambiguously identify demethylation of MeSeH as a direct pathway to H_2_Se.

### 3.7. Incorporation of Selenide into Selenosugars

Selenosugars are major urinary metabolites for Se. [110]. While many of the published metabolic pathways of Se suggest selenosugars are derived from H_2_Se, there is in fact very little information on their biosynthetic pathways, and no sound evidence that Se(−II) is directly involved [111,112].

## 4. Unanswered Questions

### 4.1. Is Se an Anti-oxidant or a Pro-Oxidant?

The answer is probably both, but the underlying mechanisms remain unresolved. The potential anti-oxidant properties of Se-containing compounds are self-evident given the chemical properties of the element, and indeed natural and synthetic Se-containing small molecules are frequently touted as potential therapies for oxidative stress [113]. Furthermore, the SeM that is randomly incorporated into proteins is thought to protect nearby residues from oxidative damage [114,115] and SeM-containing proteins are themselves thought to be anti-oxidants [113]. However, it is likely that many of the mechanisms by which Se influences redox status are not intuitive. By way of illustration, Cys serves a key role within the tripeptide glutathione (GSH) as the primary anti-oxidant in eukaryotic cells, but free Cys also acts as a reductant of Fe(III) that supports deleterious Fenton chemistry, thereby serving as a pro-oxidant [116]. Consequently, GSH is naturally maintained in a reduced state, whereas Cys is kept in an oxidized state in vivo. However, the oxidation potential of GSH is very similar to Cys [117]. Why, then, are living cells that contain millimolar concentrations of GSH oxidatively stressed upon addition of Cys [118,119,120]? One explanation could be that the kinetics of the reduction of Cys are unfavorable. Indeed, as mentioned earlier, there is evidence that the GSSG/2GSH and the CySSCy/2Cys pools are not in redox equilibrium [69]. Enter the role of Se as a chalcogen that is more kinetically labile. Selenols are known to catalyze the interchange reactions of thiols and disulfides [121]. Therefore, one can envisage a mechanism whereby Se compounds could introduce a redox imbalance and oxidative stress in vivo, e.g., via release of Cys by H_2_Se and/or selenols:

(12) RSSCy+R′SeH→RSSeR′+CySH

Alternatively, Se compounds that are capable of effecting Rxn 12 may also be capable of directly reducing Fe(III), thereby propagating the Fenton cycle.

### 4.2. Why Is Inorganic Se More Toxic Than Organic Se?

The molecular mechanism of Se toxicity remains unclear. However, there is anecdotal evidence that excess Se influences redox homeostasis, especially when the source is inorganic Se [122,123,124,125,126]. Sec and SeM, particularly in their L-enantiomers, are less toxic than SeO_3_^2−^ [127] Indeed, it is widely held that the replacement of Met with SeM in proteins does not markedly influence their structures or functions. However, as a consequence of weak Se–Se and Se–S bonds, replacement of Cys with Sec at sites of dichalogenide bonds would likely result in structural instability. Furthermore, the replacement of Cys with Sec at the active sites of enzymes is expected to influence function as a consequence of the different redox and acid/base properties of the two residues. Consistent with these potential deleterious effects of random incorporation of Sec, Cys substitution with Sec in natural selenoproteins is generally believed to be genetically encoded, although there has been a recent report of incorporation of Sec into yeast in the absence of the UGA codon [128].

So what causes the toxicity of Se? Some insight into the toxicology of Se may be garnered from studies of the anticancer properties of Se compounds [129,130,131,132,133]. Oxidative stress and apoptosis occurs when mammalian cells are treated with SeO_3_^2−^ [124,134,135,136,137,138,139,140,141,142,143]. Superoxide (O_2_^−^•) is known to be produced when SeO_3_^2−^ reacts with GSH [144]. In Vitro evidence demonstrated that non-enzymatic reduction of SeO_3_^2−^ by GSH rapidly yields GSSeSG and O_2_^−^• [135,144,145,146,147]. Additionally, apoptosis is known to be induced by ROS like O_2_^−^• [3]. While the precise mechanism by which O_2_^−^• is produced when SeO_3_^2−^ is reduced by GSH is not clear, the following stoichiometry has been proposed [147]:

(13) $${3\mathrm{SeO}}_{3}^{2^{-}}+6\mathrm{GSH}+4H^{+}\to 3\mathrm{GSSeSG}+2O_{2}^{-}•+5H_{2}O$$

In contrast, SeM is not known to produce ROS [124], and while Sec can produce ROS [124], it is more tightly regulated (*vide supra*). Although the release of ROS during the oxidation of H_2_Se has apparently not been investigated, the reaction is believed to proceed via a chain-radical mechanism, which would likely involve one-electron processes and the formation of HSe^.^ and ROS like O_2_^−^• [67]. In fact, H_2_Se breaks DNA phosphodiester bonds in vitro under aerobic conditions, and chromosome fragmentation has been observed in vivo [148,149,150], behavior that is consistent with the formation of ROS. Similar effects may be observed for SeO_3_^2−^ in the presence of reducing agents like GSH.

Besides the direct generation of ROS, it is conceivable that Se has indirect effects, for example by inhibiting thiol proteins that have roles in the management of oxidative stress. Furthermore, while the replacement of inorganic S^2−^ with Se^2−^ in vivo, for example in iron-sulfur proteins (e.g., ferredoxins), is without literature precedence, it seems logical that such substitution might be more readily achieved with inorganic sources of Se rather than organic sources like Sec or SeM. It is noteworthy that in vitro substitution of Se^2−^ for S^2−^ in ferredoxins have produced less stable proteins [151]. Since iron-sulfur proteins play critical roles in energy-related electron transfer pathways, it is possible that such substitutions might affect Fe–S/Se protein function. Surprisingly, thus far in vitro substitution of Se^2−^ for S^2−^ in Fe–S proteins has only marginally modified their properties [151]. It is noteworthy that early studies did not reveal significant differentiation between S^2−^ for Se^2−^ during chalcogenide incorporation into Fe-S proteins [151], but a recent reaction of nitrogenase with selenocyanate (*vida infra*) revealed highly selective incorporation of Se^2−^ into the FeMoCo cluster [152]. Although the current literature does not suggest that the incorporation of inorganic Se into Fe–S clusters is a major source of Se toxicity, further investigation is warranted.

### 4.3. Newly Discovered and Undiscovered Biological Selenium Compounds

It has been recently discovered that selenocyanate (SeCN^−^) is present in human fluids [102]. It is useful to discuss the possible biological significance of SeCN^−^ in parallel with its S congener thiocyanate (SCN^−^). SCN^−^ is produced endogenously as a detoxification product of the rhodanase-catalyzed reaction between cyanide (CN^−^) and thiosulfate (S_2_O_3_^2−^) in the liver [153]. In contrast, the production of SeCN^−^ apparently does not require rhodanase [102], which is consistent with the higher reactivity of selenium (*vide supra*). It has been observed that in the presence of SeO_3_^2−^, CN^−^, and GSH, SeCN^−^ is produced [102]. Higher chemical yields of SeCN^−^ are produced if SeO_3_^2−^ and GSH are replaced with selenosulfate (SeSO_3_^2−^) [102]. Given the mechanism by which rhodanase operates (reaction of Cys-247 with S_2_O_3_^2−^ to produce a persulfide, CySSH, followed by reaction with CN^−^ to regenerate Cys-247 and produce SCN^−^) [154,155], and given the higher reactivity of Se, we imagine that species like GSSeH (Equation (7)) may react with CN^−^ in vivo to produce SeCN^−^ [156]. There are many endogenous and exogenous sources of CN^−^, including the metabolism of vitamin B_12_ and certain foods containing cyanogenic glucosides, for example, nuts (especially almonds) and cruciferous vegetables (e.g., the Brassica) [157]. Nonmetabolic sources of cyanide in humans include tobacco and occupationally derived smoke (HCN, e.g., >200 mg/cigarette) [158], chlorination of glycine by human myeloperoxidase (MPO) during inflammation [159], and cyanogenesis (the biochemical production of CN^−^) by *Pseudomonas aeruginosa* (an opportunistic pathogen that infects wounds and the lungs of immune-compromised individuals) [160,161,162,163,164]. SCN^−^ is abundant in all physiologic fluids and especially in those that are derived from the mucosae (e.g., saliva, lachrymal fluids, breast milk, and the mucosal layer of the lung), where the concentration of SCN^−^ is one or two orders of magnitude larger than in blood plasma as a consequence of its active transport [165]. We note that the same transport mechanisms function with SeCN^−^ [166,167], so SeCN^−^ is apparently also concentrated in exocrine fluids [167]. Unlike many other inorganic ions, such as Ca^2+^ (biological messenger and structural component of bone), K^+^ (osmotic regulator), CO_3_^2−^ (pH buffering), and PO_4_^2−^ (ATP, DNA, etc.), SCN^−^ is not generally considered to be a biologically functional ion. This is largely because SCN^−^ is not chemically reactive (although it forms coordination-covalent bonds with metals). However, in addition to being a product of detoxification of CN^−^, the SCN^−^ ion plays an important role as a substrate for human defensive peroxidases, components of the human innate defense stratagem that include lactoperoxidase (LPO), salivary peroxidase (SPO), myeloperoxidase (MPO), and eosinophil peroxidase (EPO). The product of oxidation of SCN^−^ by the defensive peroxidases is hypothiocyanite (OSCN^−^), a non-antibiotic antimicrobial. While it is not known whether SeCN^−^ is a substrate for these aforementioned peroxidases, and while the corresponding oxidized species hyposelenocyanite (OSeCN^−^) is not known, importantly, in contrast to SCN^−^, SeCN^−^ is apparently chemically reactive, and it has been suggested that it is part of the intrinsic Se pool [102].

## 5. Conclusions

While related by the periodic table and similar chemical properties, S and Se compounds exhibit markedly different behavior in a biological setting. In general, S chemistry is regulated in vivo, whereas Se chemistry is not. Many of the chemical transformation that require enzymatic catalysts to effect for S, proceed via uncatalyzed reactions for Se. The present review has focused on the role of H_2_Se, the putative linchpin of Se biology. While it is clear that H_2_Se plays a critical role in biology, exploration of its function is hindered by low relative concentrations, high reactivity, and the fact that tell-tale cellular machinery is not required to carry out many biotransformations that likely involve H_2_Se. There is a need for a better general understanding of the chemistry of H_2_Se, and for the development of specific assays that will facilitate experimentation, including colorimetric probes of Se-containing biomolecules [8,168]

Perhaps researchers should take a cue from Nature, which has found a way to exploit the subtle physical and chemical differences of S and Se compounds, in developing novel methods to explore the biology of H_2_Se and other Se compounds.

## Figures and Tables

**Table 1:** General properties of sulfur and selenium.

Property		S	Se
Electron configuration		[Ne] 3s^2^3p^4^	[Ar] 3d^10^4s^2^4p^4^
Average atomic mass (amu)		32.06	78.96
Covalent radius (pm) [30]		100	115
van der Waals radius (pm) [31]		180	190
Bond length (pm) [32]		134 (S–H)	146 (Se–H)
		180 (S–C)	196 (Se–C)
		205 (S–S)	232 (Se–Se)
Bond energy (kJ·mol^−1^)	HX–H	381.6 (S–H) [33]	334.9 (Se–H) [34]
	CH_3_X–CH_3_	307.9 (S–C) [33]	234 (Se–C) [35]
	CH_3_X–XCH_3_	273.6 (S–S) [33]	197.6 (Se–Se) [36]
Ionization Energies (kJ·mol^−1^) [37]	1st	999.6	940.9
	2nd	2251	2045
	3rd	3361	2974
Electron affinity (kJ·mol^−1^) [38]		200	195
Pauling electronegativity [39]		2.58	2.55
Polarizability (in Å^3^)		2.90 [40]	3.89 [41]
pK_a1_, (H_2_X)		6.88 [42]	3.89 [43]
pK_a2_, (HX^−^)		14.15 [42]	15.1 [44]
pK_a2_, (Cys/Sec) [45]		8.22 [46,47]	5.43 [48]
